# Vertebrobasilar dolichoectasia to trigeminal neuralgia: case report

**DOI:** 10.1093/jscr/rjad737

**Published:** 2024-01-16

**Authors:** Fangkun Jing, Haitao Huang, Yanfeng Li, Peizhuo Zang

**Affiliations:** Department of Neurosurgery, The People's Hospital of Liaoning Province, Shenyang, Liaoning, China; Department of Neurosurgery, The People's Hospital of Liaoning Province, Shenyang, Liaoning, China; Department of Neurosurgery, The People's Hospital of Liaoning Province, Shenyang, Liaoning, China; Department of Neurosurgery, The People's Hospital of Liaoning Province, Shenyang, Liaoning, China

**Keywords:** vertebrobasilar dolichoectasia, trigeminal neuralgia, endovascular treatment

## Abstract

Vertebrobasilar dolichoectasia (VBD) is a dilated arterial disease with a diameter ˃4.5 mm. Trigeminal neuralgia (TN) is chronic neuropathic pain. Through the diagnosis and treatment of this case we believe that there is a significant risk associated with the endovascular treatment of VBD. The development of post-operative complications caused some functional impairment to the patient, but the improvement in TN symptoms with this endovascular treatment was unexpected. This treatment procedure was considered to be possibly related to the alteration of the tortuous path of the vessels, changing their course, allowing the displacement of vascular compression in the trigeminal root entry zone, and possibly also altering the hemodynamics of the posterior circulation, improving the progression of ischemia and hypoxia-induced demyelination of the trigeminal nerve. Due to the low incidence of this disease, there are not enough large sample studies for systematic statistical analysis.

## Introduction

Vertebrobasilar dolichoectasia (VBD) is a dilated arterial disease with a diameter ˃4.5 mm. It is a rare disease with progressive bulbar palsy and ataxia as the main clinical manifestations, with other cranial nerve as the first symptom [1]. There are also patients who have no clinical manifestations and are only found to have abnormal dilatation of the basilar artery during physical examination, which is reported to occur in 0.06–5.8% of the general population [2].

Trigeminal neuralgia (TN) is a chronic neuropathic pain. TN mainly presents with recurrent spontaneous or evoked paroxysmal episodes of electric shock-like or stabbing pain in the trigeminal nerve distribution on one side of the face [3, 4]. TN can be divided into primary and secondary TN, the main difference was the presence or absence of organic lesions of the trigeminal nerve, such as tumors compression, viral infections, and multiple sclerosis and so on [5].

## Case report

**Figure 1:**
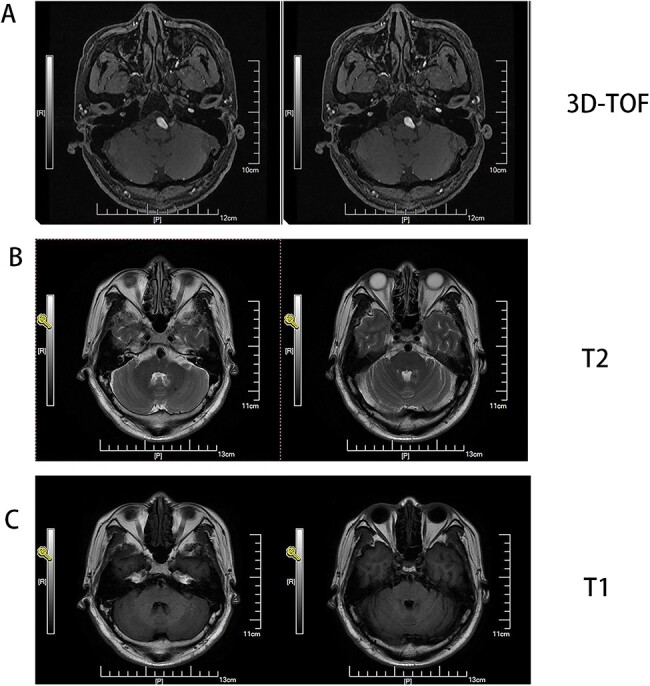
(A) MRI 3D-TOF(3 digital- time of flight), (B) MRI T2, C:MRI T1. Basilar trunk dilation up to 9 mm of diameter deviated path to the left side responsible of medulla compression.

**Figure 2:**
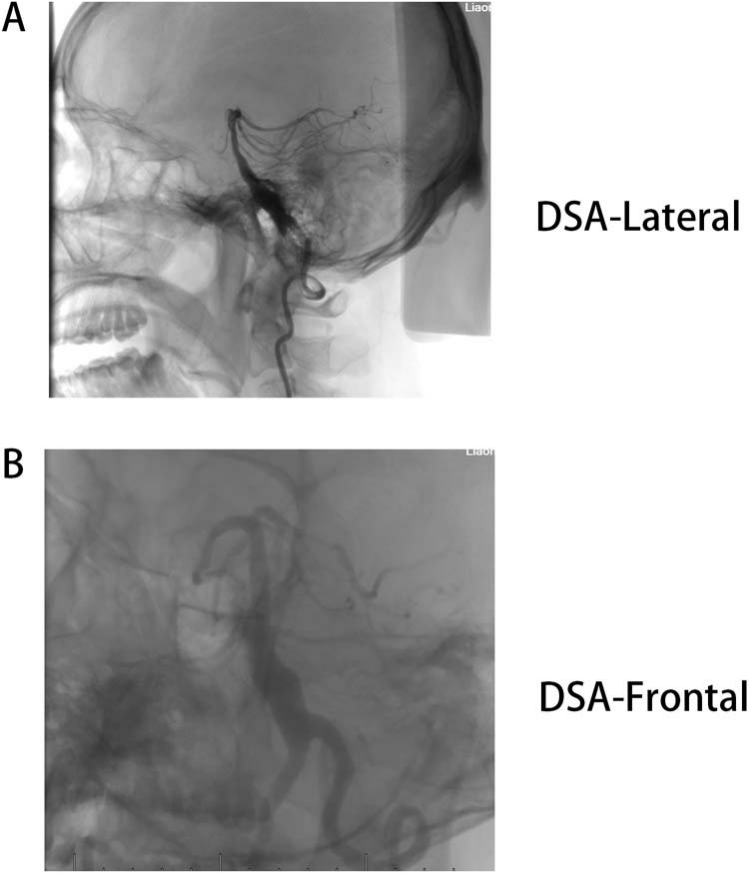
(A) DSA-lateral, (B) DSA-Frontal. Basilar trunk dilation up to 9 mm of diameter and extended over 25 mm of length with a tortuous and deviated path to the left side.

**Figure 3:**
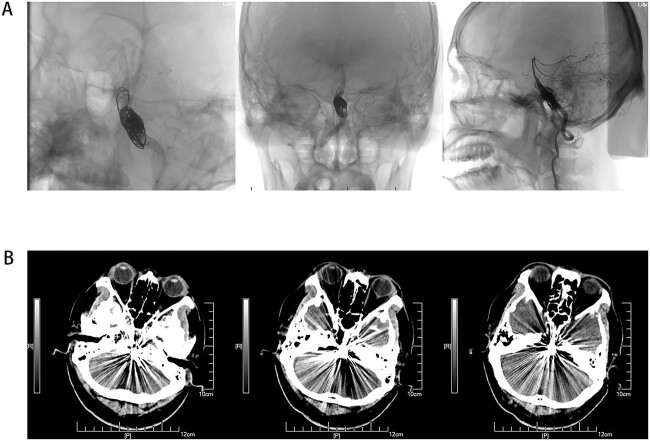
(A) Coil and stent implantation for VBD, and show peripheral blood supply after operation. (B) Non-contrast axial CT scan of the brain showing VBD after surgery.

Male, AA, 44 years old, admitted in July 2022 with recurrent pain on the left side of the maxillofacial region for ~1 year. In the last 6 months, the dose of carbamazepine was increased to 300 mg three times daily, but the control of facial pain was still unsatisfactory, so the patient and his family numbers requested to be hospitalized for surgery. The patient had a history of hypertension for ~15 years. The maximum blood pressure was ~200/120 mmHg. The nifedipine controlled-release tablet was used to control his blood pressure by 30 mg daily, which is maintained at ~140/80 mmHg. The neurological examination on admission was unremarkable. TN was assessed as grade V according to the Bar row Neurological Institute classification. Magnetic Resonance Imaging (MRI) of the head suggests small ischemic focus in the pons and cerebellum; marked extending of the basilar artery (Fig. 1A–C). To clarify the etiology, doppler ultrasound of the carotid and vertebral arteries bilaterally revealed no atheromatous plaque or hemodynamics abnormalities. An electrocardiogram and cardiac function and cardiac ultrasound were performed. The results were normal. Biochemical tests for blood cell count, liver function, kidney function, and glucose test revealed no abnormalities. The Digital subtraction angiography (DSA) was given to the patient (Fig. 2A and B). Antiplatelet aggregation therapy with a combination of clopidogrel and aspirin was used before operation. The DSA displayed the VBD, the VBD was length 2.5 cm and width 9 mm, its path was tortuous and deviated to the left. Normal blood flow in the middle venous phase of the imaging was normal. Stent-assisted interventional embolization was chosen. A suitable coil was selected to form basket by EV3 (20 mm^*^50 cm, Medtronic, USA) and the stent catheter was withdrawn and repositioned through the coil into the right posterior cerebral artery. Release stent 1: LEO (5.5 mm^*^60 mm, LEO, USA), then choose the appropriate ring (14 mm^*^30 cm, 13 mm^*^30 cm, 12 mm^*^30 cm, 10 mm^*^30 cm, Taijie Weiye, China) to occlude the dilated artery (Fig. 3A). Postoperatively, we scanned the Computed Tomography (CT) (Fig. 3A), the TN was completely relieved, but there was residual left-sided facial palsy with a House-Brackmann grade IV and a grade III right limb muscle strength. Systematic treatment was operated at the rehabilitation unit, once the condition was stabilized. At 3 months post-operative follow-up the patient showed significant improvement in muscle strength in the right limb, with muscle strength grade V^−^ and significant improvement in facial palsy, which was graded House-Brackmann grade II.

## Discussion

VBD, the risks to human health are difficult to assess and can even endanger the lives of patients. The pathophysiology of VBD is characterized by progressive dilatation of the artery due to the thinning of the underlying connective tissue within the mesentery and the fragmentation of the internal elastic layer [6]. There are many causes of vasodilation, including vascular risk factors such as age, gender, hypertension, diabetes, dyslipidemia, obesity, smoking, and so on [7]. Genetic factors including those leading to abnormal development of muscle fibers, atrophy of the smooth muscle layer, and deficiencies of the intra-arterial elastic lamina, such as autosomal inherited polycystic kidney disease, PHACES syndrome, Marfan syndrome and other diseases have been suggested to be associated with autoimmune factors and validated response factors [8]. According to the study, VBD predicts an increased risk of ischemic and hemorrhagic stroke, arterial entrapment and arterial dissection in the posterior cranial fossa. The cases were considered to be related to the patient’s previous irregular lifestyle and long history of high blood pressure in the study. Prolonged irritation of the blood vessels by high blood pressure leads to a decrease in the elasticity of the blood vessels due to a break in the connective tissue of the intima. It is also believed that the patient’s morbidity is associated with his own high basal metabolic rate and obesity. His body mass index value was 32.3.

The main treatment options for VBD include microsurgical and endovascular treatment [9, 10]. Recent advances were applied in endovascular therapy, particularly flow diverting system stents (FDSs). New hope was brought for an effective solution to the VBD problem. However, little is known about the effect of endovascular treatment (EVT) on VBD. The VBD is located mainly near the brainstem. If treatment with FDSs may cause ischemia in other important branches of the basilar artery, resulting in unpredictable outcomes. In this study, the usual endovascular stent-assisted coil implantation technique was used to treat, which allows for the definitive treatment of VBD and also reduces the occlusion of unnecessary collateral circulation, minimizing symptoms of brainstem ischemia due to occlusion of major trophoblastic vessels in the peripheral brainstem as a result of treatment modalities. It can also improve or treat other symptoms caused by tortuous blood vessels. FDSs may lead to occlusion of some branches of the basilar artery and influence patients’ quality of life. In this case, VBD was treated by endovascular therapy and TN due to tortuous or compressed vessels was also treated.

TN affects people's life and work, the main cause of primary trigeminal neuralgia is due to vascular contact with root entry zone (REZ) area of the trigeminal nerve [5]. Some studies have shown that VBD can cause symptoms by directly compressing cranial nerves or the brainstem [11]. This case is thought to be related to direct compression of the trigeminal REZ by tortuous VBD vessels and may have triggered the onset of TN due to ischemic and hypoxic induced demyelination changes in the peripheral vasculature caused by VBD, which triggered contact between the peripheral vasculature and the REZ of the trigeminal nerve. Treatment options for TN include medication, microvascular decompression (MVD), percutaneous balloon compression (PBC), percutaneous radiofrequency trigeminal gangliolysis, and so on. This patient was initially treated with carbamazepine for TN, but as the disease progressed the effectiveness of the drug treatment gradually deteriorated. We plan to apply MVD surgery before cerebrovascular examination of the head. With the return of the patient’s test results, the patient’s condition was assessed, and the choice was made to treat VBD endovascularly firstly, TN was treated by a simple and easy PBC procedure. However, the patient’s symptoms of TN improved completely after endovascular treatment with VBD and no further treatment of TN was required.

## Data Availability

The original contributions presented in the study are included in the article/figure, further inquiries can be directed to the corresponding author.
